# Detection of sleep apnea using only inertial measurement unit signals from apple watch: a pilot-study with machine learning approach

**DOI:** 10.1007/s11325-025-03255-w

**Published:** 2025-02-01

**Authors:** Junichiro Hayano, Mine Adachi, Yutaka Murakami, Fumihiko Sasaki, Emi Yuda

**Affiliations:** 1 Department of Research and Development, Heart Beat Science Lab Inc., Nagoya, Japan; 2 Takaoka Clinic, Nagoya, Japan; 3 Sony Group Inc, Tokyo, Japan; 4 Graduate School of Information Sciences, Tohoku University, Sendai, Japan

**Keywords:** Acceleration, Apnea-hypopnea index, Gyrocardiogram, Gyroscope, Home sleep apnea testing, Out-of-center sleep testing, Seismocardiogram, Wearable sensor

## Abstract

**Purpose:**

Despite increased awareness of sleep hygiene, over 80% of sleep apnea cases remain undiagnosed, underscoring the need for accessible screening methods. This study presents a method for detecting sleep apnea using data from the Apple Watch’s inertial measurement unit (IMU).

**Methods:**

An algorithm was developed to extract seismocardiographic and respiratory signals from IMU data, analyzing features such as breathing and heart rate variability, respiratory dips, and body movements. In a cohort of 61 adults undergoing polysomnography, we analyzed 52,337 30-second epochs, with 12,373 (23.6%) identified as apnea/hypopnea episodes. Machine learning models using five classifiers (Logistic Regression, Random Forest, Gradient Boosting, k-Nearest Neighbors, and Multi-layer Perceptron) were trained on data from 41 subjects and validated on 20 subjects.

**Results:**

The Random Forest classifier performed best in per-epoch respiratory event detection, achieving an AUC of 0.827 and an F1 score of 0.572 in the training group, and an AUC of 0.831 and an F1 score of 0.602 in the test group. The model’s per-subject predictions strongly correlated with the apnea-hypopnea index (AHI) from polysomnography (*r* = 0.93) and identified subjects with AHI ≥ 15 with 100% sensitivity and 90% specificity.

**Conclusion:**

Utilizing the widespread availability of the Apple Watch and the low power requirements of the IMU, this approach has the potential to significantly improve sleep apnea screening accessibility.

## Introduction

Sleep apnea, a prevalent sleep disorder, has garnered considerable attention due to its association with daytime drowsiness, cognitive impairment, and cardiovascular diseases [1, 2]. Despite this, over 80% of cases remain undiagnosed [3], creating a substantial burden on healthcare systems [4, 5]. This underdiagnosis is primarily due to a lack of awareness among patients about the need for testing unless symptoms become severe, and even simplified home sleep apnea testing (HSAT) tools are underutilized [6]. To address this issue, it is essential to develop screening methods that increase opportunities for screening for sleep apnea detection without relying on patient awareness.

In this context, the potential use of smartwatches as accessible screening tools for sleep apnea has gained considerable interest [7–12]. Most studies reported a good agreement between the frequency of respiratory events (REs) detected by smartwatches and the apnea-hypopnea index (AHI) derived from polysomnography (PSG), the gold standard method. However, these studies typically report only whole-night summary statistics without providing detailed epoch-by-epoch or event-by-event analyses. Consequently, even if comparable metrics are achieved between smartwatch methods and PSG, they may be based on labeling and scoring different events [13]. To fully evaluate the potential and limitations of smartwatches as a screening tool for sleep apnea, it is crucial to identify the optimal signals and feature sets associated with REs.

This study examines the feasibility of detecting REs of sleep apnea using acceleration and gyroscope signals from the inertial measurement unit (IMU) embedded in Apple Watches^®^. While IMUs in smartwatches are primarily utilized for assessing physical activity [14, 15], they can also detect subtle movements caused by heartbeat and respiration [16], even when worn on the wrist. In previous work [17], we demonstrated that the frequency of transient dips in the amplitude of respiratory motion detected by an IMU correlates with the AHI in patients with suspected sleep apnea. Expanding on this, the present study explored the optimal feature set of IMU signals and model to detect REs of sleep apnea. Considering the influence of individual factors and device positioning on inertial forces, we introduced novel features for RE detection that are independent of absolute signal strength. By leveraging machine learning to model these complex relationships, our findings reveal that these IMU-derived features enhance both per-epoch classifications of REs and the accuracy of per-subject AHI estimation.

## Methods

### Ethics approval and consent to participate

The study was conducted following the ethical guidelines issued by the Japanese Ministry of Health, Labor and Welfare, the 1964 Declaration of Helsinki, and its amendments. The protocol was approved by the Research Ethics Committee of the Graduate School of Information Sciences, Tohoku University, Japan (approved number 23 A-02). Written informed consent was obtained from all subjects.

### Subjects

Participants were adults who underwent overnight PSG at Takaoka Clinic, Nagoya, Japan, between August 25, 2023, and November 13, 2023. Inclusion criteria were age 20 years or older, with exclusions for acute illness or recent hospitalization, pregnancy, or breastfeeding.

### Protocol

Subjects stayed overnight in a PSG testing chamber equipped with an Embla N7000 PSG amplifier. They wore an Apple Watch SE (40 mm, version 9.5.2, 20T571, model A2722, Apple Inc, Cupertino, California, USA)) on their left wrist to continuously record 3-axis acceleration and gyroscope signals from its inertial measurement unit (IMU). Data were transferred to a secure cloud environment via a smartphone (iPhone^®^, model MMYD3J/A, iOS version 16.6.1, Apple Inc) connected to the watch.

### Measurements

PSG recordings included standard electroencephalograms, electrooculograms, electromyograms, respiratory sensors, and a modified electrocardiogram. Sleep-disordered breathing events were scored based on the American Academy of Sleep Medicine (AASM) Manual for the Scoring of Sleep and Associated Events, version 2.5 [18]. The severity of sleep apnea was classified as follows: AHI < 5 was classified as normal, 5–15 as mild, 15–30 as moderate, and 30 or more as severe. IMU data were sampled at 60 Hz. The resolutions of acceleration and gyroscope were 0.0153 mG and 0.0000153 degrees per sec (dps), respectively. Synchronization between IMU and PSG data was achieved using timestamps.

### Signal processing and feature extraction

**Fig. 1:**
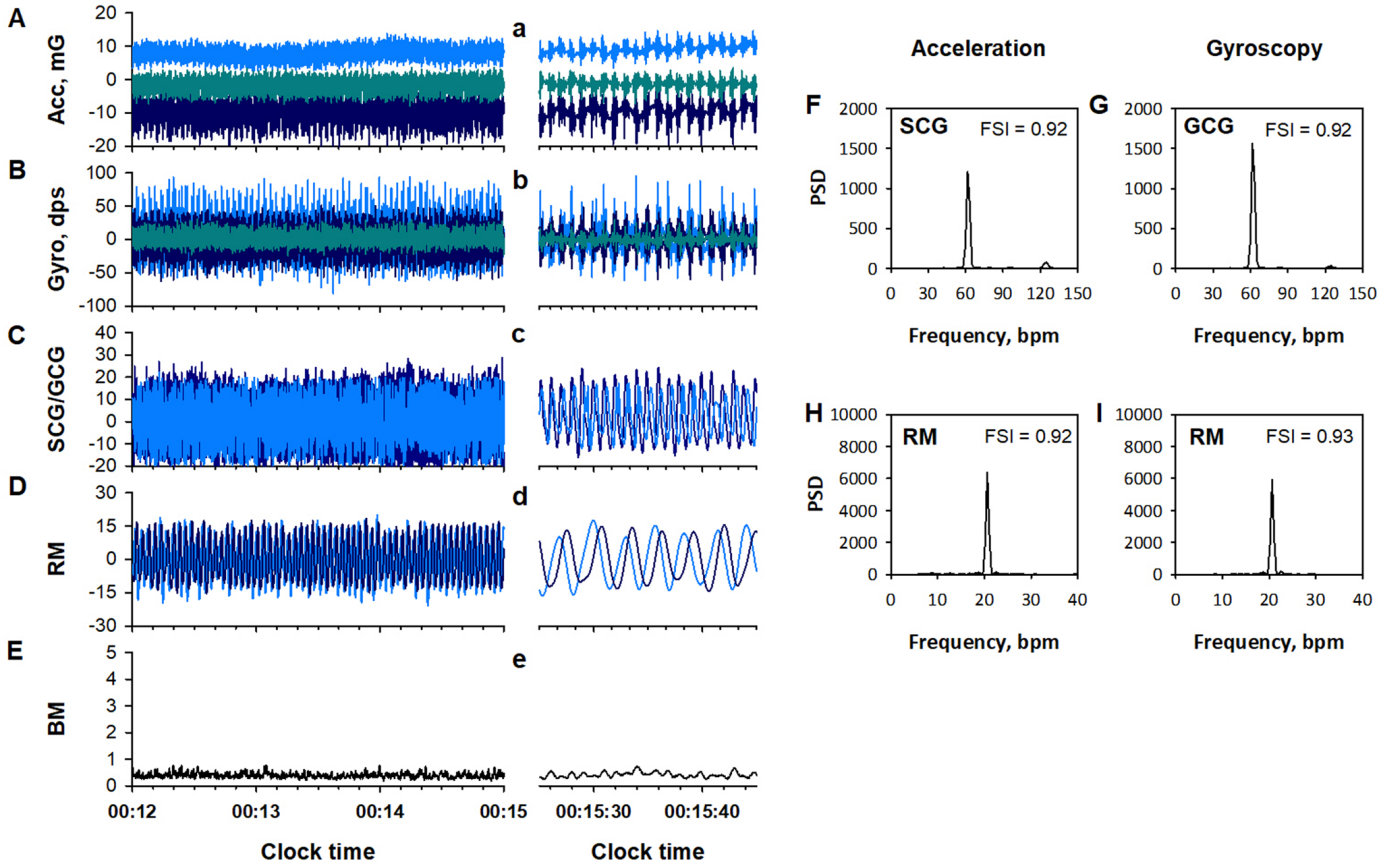
Examples of Apple Watch’s inertial measurement unit (IMU) data (**A**, **B**), their component signals (**C**-**E**), and power spectra (**F**-**I**) during normal breathing in a typical subject’s sleep. Dark blue, blue, and dark cyan lines in panels A and B represent X-, Y-, Z-axis data, respectively, of acceleration (acc, panel A) and gyroscope (gyro, panel B). Dark blue and blue lines in panel C represent seismocardiogram (SCG) and gyrocardiogram (GCG) components from acceleration and gyroscope data, respectively. Dark blue and blue lines in panel D represent respiratory wrist motion (RM) components from acceleration and gyroscope data, respectively. Panel E shows gross body movement (BM) component. The lower-case panels show the data from each of the upper-case panels in an enlarged time frame. Panels F to I show the fast Fourier transform power spectra of the SCG, GCG, acceleration RM, and gyroscope RM, respectively. The frequency stability index (FSI) within these panels is described in Fig. 3

**Fig. 2:**
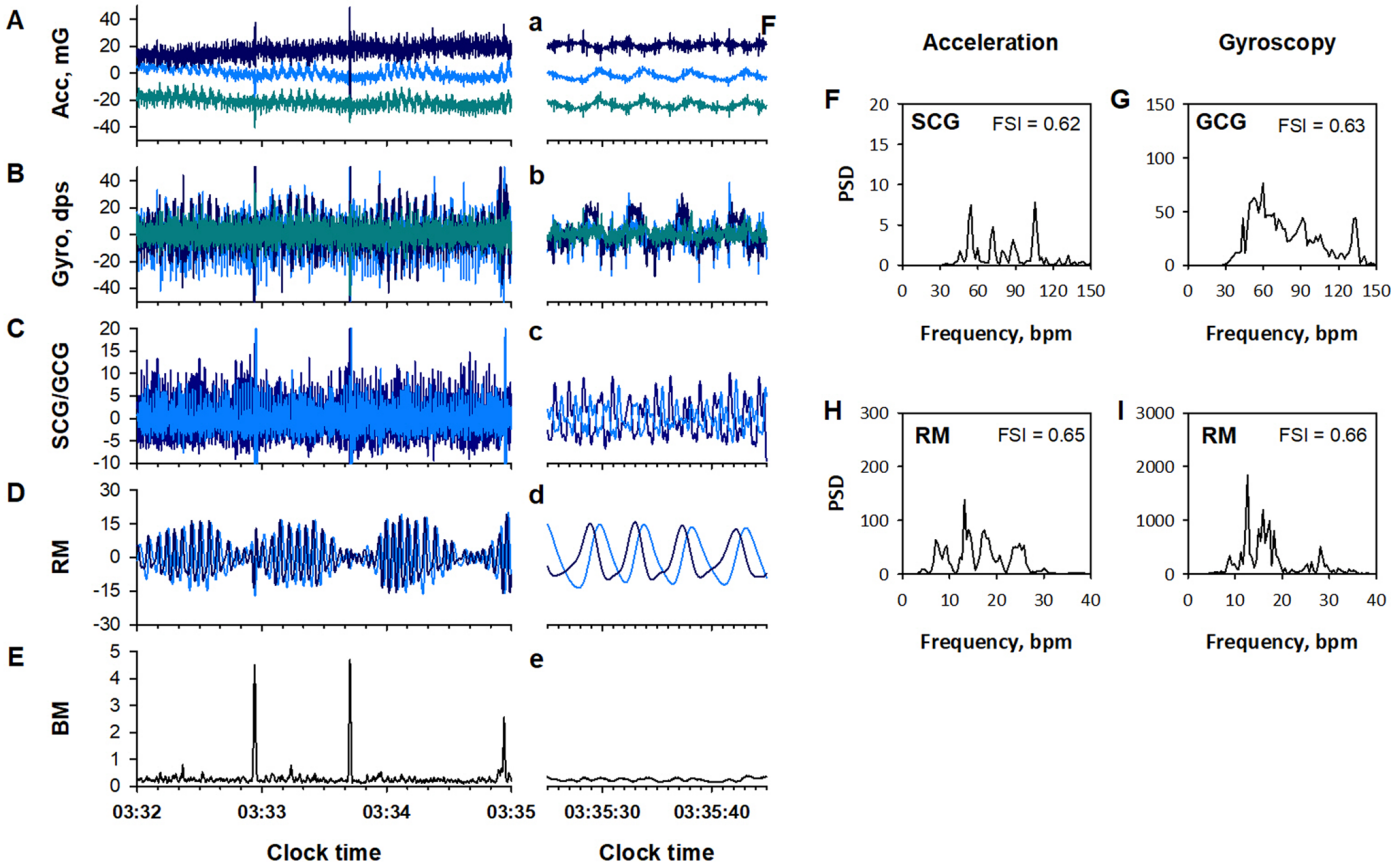
Examples of Apple Watch’s IMU data (**A**, **B**), their component signals (**C**-**E**), and power spectra (**F**-**I**) during sleep disordered breathing in a typical subject’s sleep. Dark blue, blue, and dark cyan lines in panels A and B represent X-, Y-, Z-axis data, respectively, of acceleration (acc, panel A) and gyroscope (gyro, panel B). Dark blue and blue lines in panel C represent seismocardiogram (SCG) and gyrocardiogram (GCG) components from acceleration and gyroscope data, respectively. Dark blue and blue lines in panel D represent respiratory wrist motion (RM) components from acceleration and gyroscope data, respectively. Panel E shows gross body movement (BM) component. The lower-case panels show the data from each of the upper-case panels in an enlarged time frame. Panels F to I show the fast Fourier transform power spectra of the SCG, GCG, acceleration RM, and gyroscope RM, respectively. The frequency stability index (FSI) within these panels is described in Fig. 3

An algorithm was developed to extract features from IMU data reflecting mechanocardiogram (MCG), respiratory wrist motion (RM), and gross body movement (BM) to detect sleep apnea/hypopnea episodes (Figs. 1 and 2).

### Mechanocardiogram (MCG)

MCG is a physiological measure of body surface vibration linked to cardiac cycle, including seismocardiogram (SCG) and gyrocardiogram (GCG), which are measured with an accelerometer and a gyroscope, respectively [19, 20]. To extract the MCG components from acceleration and gyroscope data, the X-, Y-, and Z-axis signals of each data were processed separately with a finite impulse response (FIR) band-pass filter set at 4–11 Hz (panels C and c in Figs. 1 and 2). The filtered triaxial signals of acceleration and gyroscope were combined into scalar time series.

To quantify the disruption of SCG and GCG frequency structures with sleep apnea (panels Fand G in Fig. 2), a frequency stability index (FSI) was introduced (Appendix). FSIs for SCG and GCG were calculated for every 32-sec-long segment, moving in 30-sec steps and overlapping by 2 s at each end. Since our goal was to capture MCG instability due to sleep apnea episodes, we compared the FSIs of SCG and GCG signals for each epoch and adopted the larger of the two as the FSI value of MCG (MCG_FSI) for that epoch.

### Respiratory wrist motion (RM)

IMU data also include RM component [16, 17]. To extract the RM component from acceleration and gyroscope data, the X-, Y-, and Z-axis signals of each data were processed with an FIR band-pass filter set at 0.13–0.70 Hz. The RM components of acceleration and gyroscope were analyzed separately.

The RM components were processed in two ways. First, the X-, Y-, Z-axis data were divided into 30-s segments. In each segment, the data with the largest interquartile (25th-75th percentiles) width among X, Y, and Z axes was selected for that epoch (panel D in Figs. 1 and 2). The disruption of RM signal frequency structure (panels H and I in Fig. 2) was quantified with FSI. The FSI was calculated for every 64-sec-long segment, moving in 30-sec steps and overlapping by 34 s at each end. The FSI of acceleration and gyroscope RM signals were compared for each 30-sec epoch, and the larger of the two was adopted as the FSI value (RM_FSI) for that epoch.

**Fig. 3:**
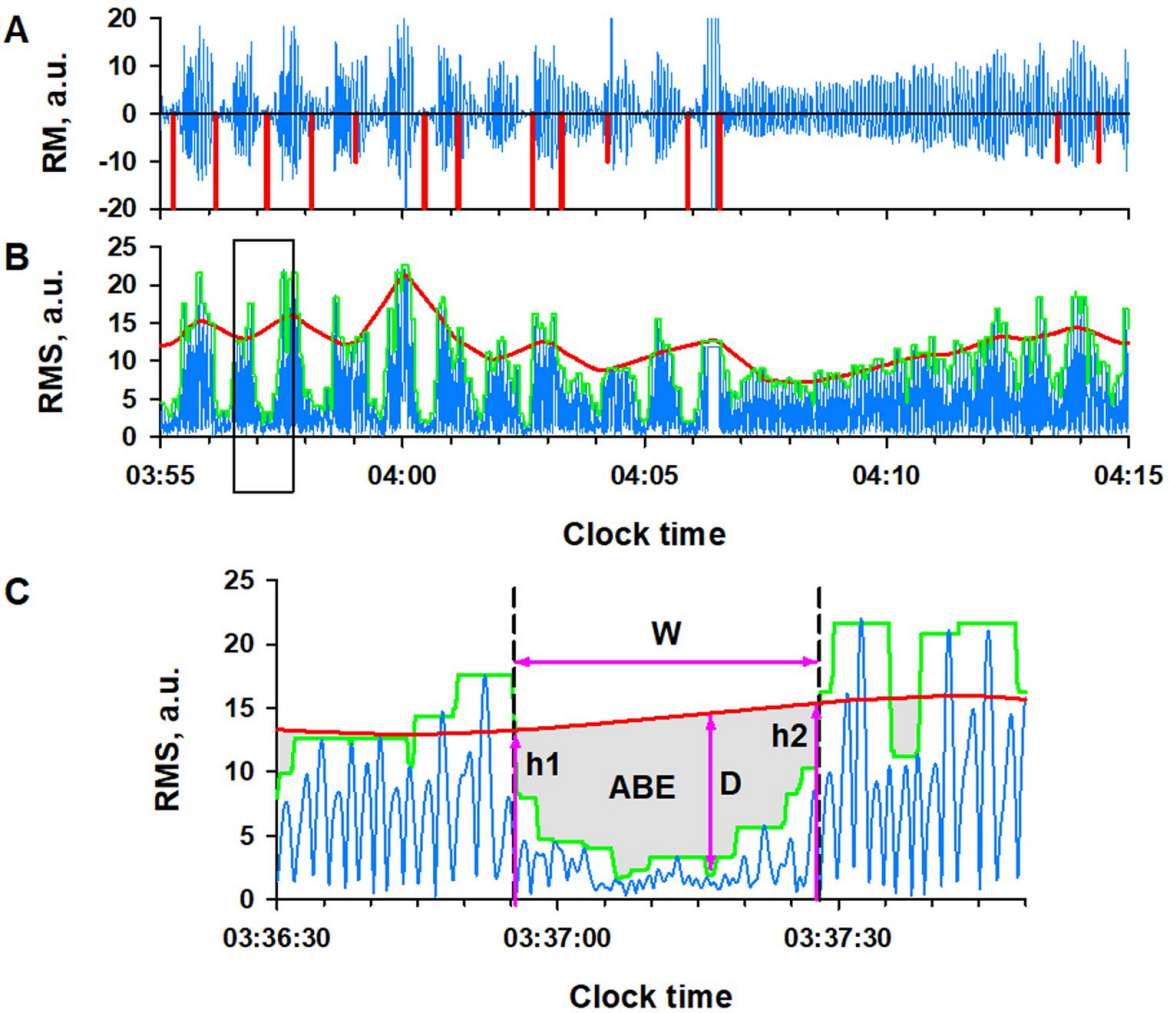
Extraction of RMS dip features. In panel **A**, the blue line represents RM signal obtained from IMU’s Acc data, while the long and short red bars denote the positions of sleep apnea and hypopnea, respectively, detected by PSG. In panel **B**, the blue line represents the scalar time series of RM (RMS) obtained from respiratory components on X-, Y-, and Z-axis of acceleration data. The green and red lines represent the fast and slow upper envelopes, respectively, calculated as 95th-percentile values within moving windows of 3- and 20-sec width, respectively. Panel **C** shows an expanded view of the squared area in panel B, demonstrating the features to utilized to characterize transient decreases in RMS, termed “RMS dips”, defined as periods when the fast envelope falls below the slow envelope. rABE denotes the area between the fast and slow envelopes relative to the area under the slow envelope, W represents the width of the RMS dip, D signifies the maximum depth of the RMS dip, and h1 and h2 correspond to the heights of the envelopes at the beginning and end of the RMS dip, respectively

Second, the filtered triaxial RM components were combined into scalar time series (RMS) (Fig. 3). According to a previous study [17], the fast and slow envelopes of RMS were calculated as 95th-percentile values within moving windows of 3- and 20-sec width, respectively (Fig. 3B). Transient dips in RMS were detected as periods when the fast envelope dropped below the slow envelope (Fig. 3C). The characteristics of each RMS dip were quantified using six features: width (W), area between the slow and fast envelopes relative to area under the slow envelope (rABE), the height of the envelopes at the beginning and end of the RMS dip (h1 and h2), and the maximum depth in absolute value (aD) and relative value to the slow envelope (rD). The h1 and h2 were converted to h_max, the higher value between h1 and h2. The features of RMS dips were calculated for both acceleration and gyroscope RM components.

### Gross body movement (BM)

BM component was extracted from the IMU acceleration and gyroscope signals (panels E and e in Figs. 1 and 2). The X-, Y-, and Z-axes data of acceleration and gyroscope were processed with band-pass filter set at 2.0–3.0 Hz. The triaxial BM components from both the acceleration and gyroscope were combined into single scalar and the maximum value in each 30-sec epoch was adopted as the BM feature of that epoch.

### Machine learning

#### Machine learning setup

We developed machine learning models to identify 30-second epochs containing respiratory events (REs), defined as apnea/hypopnea episodes identified by PSG. Each epoch was labeled as either RE-positive or RE-negative. A feature dataset was created, consisting of 15 features including MCG_FSI, RM_FSI, six features of RMS dips in acceleration and gyroscope signals, and maximum BM values during the epoch.

RMS dips were considered if the dip ended within the epoch; in cases with multiple dips, the one with the greatest ABE was used. If no dips were identified, specific features were set to zero. We aligned PSG-derived apnea/hypopnea episodes with RMS dip features within the same 30-second epoch to account for any time discrepancies.

### Data partitioning

Subjects were divided into training (67%) and test (33%) groups, ensuring balanced sleep apnea severity between groups. All data were pooled into respective training and test datasets containing feature values and ground-truth labels. There was no missing data in either dataset.

### Variable selection and model training

Machine learning was conducted on a PC with Core i9 processors using R (version 4.4.0) and RStudio. Variable selection was performed using Recursive Feature Elimination (RFE) in the *caret* library package [21] with a Naïve Bayes classifier and bootstrap resampling to ensure result generalizability. Five classifiers were trained: Logistic Regression, Random Forest, Gradient Boosting Machine, k-Nearest Neighbor, and Multilayer Perceptron, using the caret package. To address class imbalance, Synthetic Minority Oversampling (SMOTE) was applied, and hyperparameters were automatically tuned.

### Model training evaluation and final model selection

Model performance was assessed on the training dataset based on per-epoch classification and per-subject apnea-severity estimation. For per-epoch classification performance, metrics included area under the receiver operating characteristic curve (AUC), accuracy, sensitivity, specificity, positive predictive accuracy (PPA), negative predictive accuracy (NPA), and F1 score. For per-subject apnea-severity estimation performance, the correlation between the hourly frequency of RE-positive epochs, respiratory event index (REI), and PSG-derived AHI were evaluated. The final model was chosen based on its overall performance across these metrics.

### Validation in the test group

The final model’s performance was validated using the test group, with evaluation parameters identical to those in the training phase. Feature importance in the final model was determined using the *varImp* function from the caret library.

### Statistical analysis

Differences in quantitative and categorical variables between two groups were assessed using the Wilcoxon rank sum test and χ2 test, respectively. The correlation between two variables were examined through linear regression analysis and Pearson’s correlation coefficient. Statistical significance was defined as *P* < 0.05.

## Results

### Subjects’ characteristics

A total of 68 patients were enrolled, with IMU data transfer failures occurring in 7 cases. The remaining 61 patients (7 females, 11%) were included in the analysis. The median age was 50 years (IQR: 45–57), and the median body mass index (BMI) was 25.3 kg/m² (IQR: 24.2–27.2). PSG results showed a median AHI of 14.1 (IQR: 7.6–28.6), with 48% of subjects classified as having moderate-to-severe sleep apnea (AHI ≥ 15) and 25% as having severe sleep apnea (AHI ≥ 30). Obstructive, central, and mixed apnea episodes comprised 79%, 17%, and 4% of the total, respectively.

Patients were semi-randomly divided into training (41 subjects) and test (20 subjects) groups to ensure balanced sleep apnea severity between groups (Table 1). No significant differences were observed in demographic or clinical parameters between the two groups.

### Datasets for machine learning

A total of 52,337 epochs were analyzed, with 35,003 epochs in the training group and 17,334 in the test group. Of these, 22.9% of the training group and 25.2% of the test group epochs were labeled as RE-positive based on PSG results.

### Model training and evaluation

**Fig. 4:**
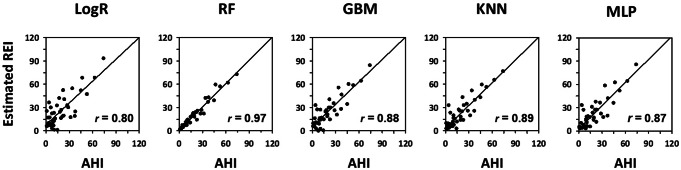
Correlation of respiratory event index (REI) estimated by candidate models with PSG-derived AHI in the training group (per-subject apnea-severity estimation performance). LogR, logistic regression; RF, Random Forest; GBM, Gradient Boosting Machine; KNN, k-Nearest Neighbor; MLP, Multilayer Perceptron

Variable selection using Recursive Feature Elimination indicated that maximum classification accuracy was achieved with all 15 features (Appendix Fig. 7). Five models were trained, with Random Forest and Gradient Boosting Machine showing the best per-epoch classification performance (Table 2). Random Forest also demonstrated the highest correlation with AHI for per-subject apnea-severity estimation (Fig. 4 and Appendix Table 2), and it was selected as the final model.

### Validation in the test group

**Fig. 5:**
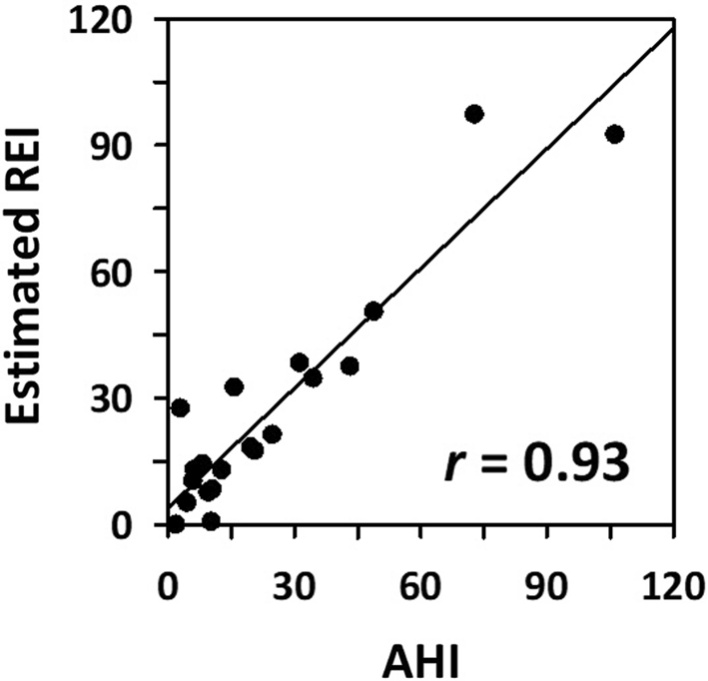
Correlation of REI estimated by the selected final model (Random Forest) with PSG-derived AHI in the test group (per-subject apnea-severity estimation performance)

Applying the Random Forest model to the test dataset showed similar classification performance to the training dataset (Table 3), indicating minimal overfitting. The correlation coefficient between the REI estimated by the model and PSG-derived AHI was 0.93 (Fig. 5 and Appendix Table 4). The model identified moderate-to-severe sleep apnea with 100% sensitivity and 90% specificity, and severe sleep apnea with both 100% sensitivity and specificity.

### Feature importance

In the Random Forest model, the most important features for RE classification were the FSI of RM, BM, and MCG FSI, followed by acceleration and gyroscope h_ratio (Appendix Fig. 8). Other RMS dip features had relatively lower importance.

## Discussion

This study developed a method to detect sleep apnea using only acceleration and gyroscope data from the Apple Watch’s built-in IMU sensor. Our approach utilized machine learning models to detect apnea/hypopnea episodes, with a Random Forest model ultimately chosen as the best-performing algorithm. Validation in the test group showed that the model’s per-subject apnea-severity estimation (REI) had a strong correlation with the AHI (*r* = 0.93) and identified moderate-to-severe sleep apnea with 100% sensitivity and 90% specificity, demonstrating promising accuracy.

The field of HSAT has seen significant advancements, with various sensors and indicators being developed to detect sleep apnea. These include nasal pressure/temperature sensors [22], respiratory inductance plethysmography [23], peripheral arterial tonometry [24, 25], oximetry [26], cyclic variation of heart rate derived from electrocardiography [27], radio-wave Doppler effect [28], and bed-embedded micromotion sensors [29–31]. While HSATs play a vital role in mitigating the limited availability of PSG, they remain underutilized despite costing less than one-fifth of PSG and being covered by health insurance schemes [6]. This underutilization appears to stem from a more fundamental issue: the lack of disease awareness among the majority of patients.

To bridge this gap and direct undiagnosed patients to clinical care, smartwatches have emerged as a promising tool, leveraging a variety of technologies for sleep apnea detection. These include photoplethysmographic (PPG) pulse rate variation [32], PPG-based pulse oximetry [8, 9], and multi-modal analyses combining PPG and accelerometer data [10–12]. IMU sensors have also been explored for respiratory monitoring in at least two previous studies [16, 17]. However, the present study is distinct in its approach, fully utilizing the IMU embedded in a widely available consumer device, the Apple Watch, for detecting sleep apnea.

In this study, we employed supervised machine learning to develop a model for the quantitative detection of sleep apnea using features derived from IMU data. The model was designed to classify epochs as either RE-positive or RE-negative based on 15 features per epoch. Given the anticipated redundancy and complex interactions among these features, machine learning was deemed the most suitable approach. To mitigate the risk of overfitting, which can compromise the model’s generalizability, we employed the Recursive Feature Elimination method to discard ineffective features and performed 25-time bootstrap resampling on the training data. This process validated the relevance of all 15 features, leading to a model that demonstrated strong performance, with minimal differences in classification metrics between the training and test groups, indicating no overfitting.

To identify the best model and evaluate its effectiveness, we assessed both per-epoch classification performance and per-subject apnea-severity estimation. The results indicated that per-epoch classification performance was modest compared to per-subject apnea-severity estimation. This modest performance may be attributed to the way PSG’s REs are assigned to epochs: since REs often span multiple epochs, the associated changes in IMU data features may not be confined to RE-positive epochs. This mismatch likely reduces the model’s sensitivity and specificity at the per-epoch level. However, the strong performance in per-subject apnea-severity estimation could be explained by the offsetting of false-negative and false-positive detections within each subject.

In the final Random Forest model, the FSI of RM emerged as the most significant factor in per-epoch classification. This may be because the RM FSI is calculated using a 64-second moving window, which potentially accommodates the reduced detection sensitivity caused by epoch labeling inconsistencies. Additionally, since the REs of PSG often label the epoch containing the end of each RE as positive, RM spectral disturbances are more likely to be captured when breathing resumes at the conclusion of an RE.

BM was the second most influential feature, likely because it helps differentiate changes in the RMS due to actual REs from those caused by large body motions. Large body movements can induce significant transient fluctuations in the IMU data within the frequency bands of both RM and BM components, leading to apparent dips in RMS FSI. BM features are crucial in distinguishing these motion artifacts from sleep apnea episodes, which also explains why h_ratio_gyr and h_ratio_acc were the strongest contributors among the RMS dip features.

The hourly frequency of RE-positive epochs estimated by the final model showed a strong correlation with PSG-derived AHI (*r* = 0.93). In the test group, the model identified subjects with moderate-to-severe sleep apnea with 100% sensitivity and 90% specificity, and those with severe sleep apnea with 100% accuracy. This performance matches or exceeds that of Type 3 HSAT devices as reported in the American Academy of Sleep Medicine’s clinical guidelines, where six devices tested in 457 participants had sensitivity ranging from 62 to 94% and specificity from 25 to 97% [33]. Remarkably, this high performance was achieved using only a single sensor unit from the Apple Watch’s built-in IMU.

With over 80% of sleep apnea cases remaining undiagnosed, this condition poses a significant societal and medical burden, increasing the risk of accidents, cognitive impairment, and cardiovascular diseases [2, 34]. Many individuals with symptoms like insomnia or daytime sleepiness often lack access to sleep testing, even with HSAT devices, unless sleep apnea is specifically suspected. Leveraging the widespread use of the Apple Watch for sleep apnea detection could greatly expand screening opportunities for these patients. IMUs consume less power compared to PPG sensors, which makes them more suitable for long-term continuous monitoring—a key factor in effective sleep apnea detection [32, 35]. Our approach, utilizing the Apple Watch’s built-in IMU, holds promise as an accessible and efficient tool for improving sleep apnea management.

This study has several limitations. The model’s performance was validated only internally, as the test group data were collected in the same clinic and under the same conditions as the training group. External validation is necessary to confirm its effectiveness as an HSAT tool. The study subjects were patients undergoing PSG for sleep apnea diagnosis or treatment evaluation, with a 50% pretest probability of moderate-to-severe sleep apnea. In a population with a lower pretest probability, the model’s sensitivity and accuracy might decrease. Additionally, the model’s performance could be influenced by patient comorbidities and the type of sleep apnea (obstructive, central, or mixed). Future research should explore the impact of these factors and validate the method’s applicability in diverse, real-life settings.

## Conclusions

We developed a feature extraction algorithm and machine learning model capable of estimating sleep apnea severity using only acceleration and gyroscope signals from the Apple Watch’s built-in IMU sensor. Leveraging the widespread adoption of the Apple Watch and the IMU’s low power consumption, this approach has strong potential to improve accessibility to sleep apnea screening.

**Table 1:** Subjects’ characteristics in the training and test groups

	Training group *N* = 41	Test group *N* = 20	P*
Age, year	51 (45–55)	49 (43–58)	0.7
Female, n (%)	6 (15%)	1 (5%)	0.4
BMI, kg/m2	25.2 (23.3–26.9)	25.9 (24.3–29.0)	0.3
Purpose of PSG			0.7
Diagnosis, n(%)	27 (66%)	15 (75%)	
CPAP titration, n(%)	10 (24%)	4 (20%)	
Other, n(%)	4 (10%)	1 (5%)	
Total recording time, min	513 (498–533)	510 (503–531)	0.9
Total sleep time, min	392 (306–417)	393 (338–422)	0.9
Sleep efficiency, %	78.4 (67.2–84.6)	78.7 (71.3–82.1)	0.8
AHI	14.1 (7.6–27.4)	14.2 (7.2–32.8)	0.7
AI	3.1 (1.1-9.0)	4.4 (1.3–15.8)	0.4
HI	8.5 (4.1–15.8)	8.0 (4.6–22.4)	0.7
OAI	1.3 (0.1–5.8)	2.5 (0.1–11.2)	0.4
CAI	0.4 (0.1–1.3)	0.5 (0.2–0.8)	0.7
MAI	0.0 (0.0-0.4)	0.1 (0.0-0.5)	0.1
AHI ≥ 15	19 (46%)	10 (50%)	0.7
AHI ≥ 30	9 (22%)	6 (30%)	0.4

**Notes**: Data are median (IQR) or frequency (%)

*Significance of difference by Wilcoxon rank sum test for continuous variables or Chi-square test for categorical variables

**Abbreviations**: AHI, apnea-hypopnea index; AI, apnea index; CAI, central apnea index; HI, hypopnea index; BMI, body mass index; CPAP, continuous positive airway pressure; MAI, mixed apnea index; OAI, obstructive apnea index; PSG, polysomnography

**Table 2:** Per-epoch classification performance of candidate models for classifying RE-positive and RE-negative epochs in the training dataset

Model	AUC	Accuracy	Sensitivity	Specificity	PPA	NPA	F1_Score
Logistic regression	0.750	0.695	0.669	0.702	0.400	0.877	0.500
Random Forest	0.827	0.779	0.646	0.818	0.513	0.886	0.572
Gradient Boosting Machine	0.828	0.750	0.732	0.756	0.470	0.905	0.573
k-Nearest Neighbor	0.768	0.698	0.706	0.696	0.408	0.889	0.517
Multilayer Perceptron	0.805	0.725	0.728	0.724	0.439	0.900	0.548

**Abbreviations**: AUC, area under the receiver-operating characteristic curve; NPA = negative predictive accuracy; PPA = positive predictive accuracy; RE, respiratory event

**Table 3:** Per-epoch classification performance metrics for the selected final model (Random Forest) at training and testing

Metric	Dataset	
	Training	Testing
	41 subjects	20 subjects
	35,003 epochs	17,334 epochs
AUC	0.827	0.831
Accuracy	0.779	0.774
Sensitivity	0.646	0.677
Specificity	0.818	0.807
PPA	0.513	0.542
NPA	0.886	0.881
F1Score	0.572	0.602

Abbreviations are explained in the footnote to Table 2

## Appendix

### Frequency stability index (FSI)

**Fig. 6:**
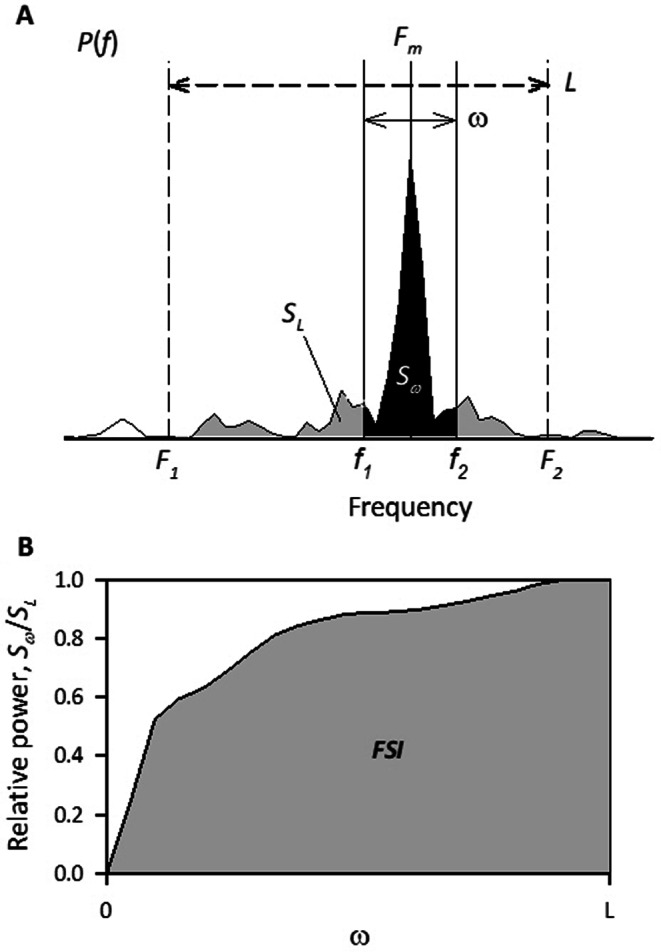
Computation of frequency stability index (FSI)

FSI measures how concentrated the power of an oscillatory signal is around its highest peak in the power spectrum (Fig. 6). It indicates the stability of the signal’s frequency.

Let *P*(*f*) be the power spectrum of the signal with the highest spectral peak at *F*_*m*_ (panel A of Fig. 6), let the analysis frequency band of that signal be *F*_*1*_ to *F*_*2*_, and let *S*_*L*_ be the power of *P*(*f*) between the two,

$$\:{S}_{L}={\int\:}_{{F}_{1}}^{{F}_{2}}P\left(f\right)\:df$$

where $\:L={F}_{2}-{F}_{1}$. Now, let $\:U=\text{m}\text{a}\text{x}({F}_{m}-{F}_{1},\:{F}_{2}-{F}_{m})$ and let *u* be a mediating variable varying from 0 to *U*,

$$\:{f}_{1}=\text{max}\left({F}_{1},\:{F}_{m}-u\right)\:\:\:(0<u<U)$$

$$\:{f}_{2}=\text{min}\left({F}_{m}+u,\:{F}_{2}\right)\:\:\:(0<u<U)$$

Then, let *S*_*ω*_ be the power of *P*(*f*) between *f*_*1*_ and *f*_*2*_.

$$\:{S}_{\omega\:}={\int\:}_{{f}_{1}}^{{f}_{2}}P\left(f\right)\:df\:\:\:(0<\omega\:<L)$$

Where $$\:\omega\:={f}_{2}-{f}_{1}$$.

Let *R*_*ω*_ be the ratio of *S*_*ω*_ to *S*_*L*_.

$$\:{R}_{\omega\:}=\frac{{S}_{\omega\:}}{{S}_{L}}$$

Then, FSI is defined as the area under the curve (AUC) of *R*_*ω*_ (panel B of Fig. 6).

$$\:\text{F}\text{S}\text{I}=\:\frac{1}{L}{\int\:}_{0}^{L}{R}_{\omega\:}d\omega\:$$

FSI ranges from 0 to 1, indicating how concentrated the power of a signal is near its highest peak in the power spectrum. An FSI of 1.0 signifies all power is concentrated at a single frequency, 0.5 indicates an even distribution across frequencies, and values approaching 0 reflect power dispersed away from the peak.

### Variable selection for classification models by recursive feature elimination (RFE)

**Fig. 7:**
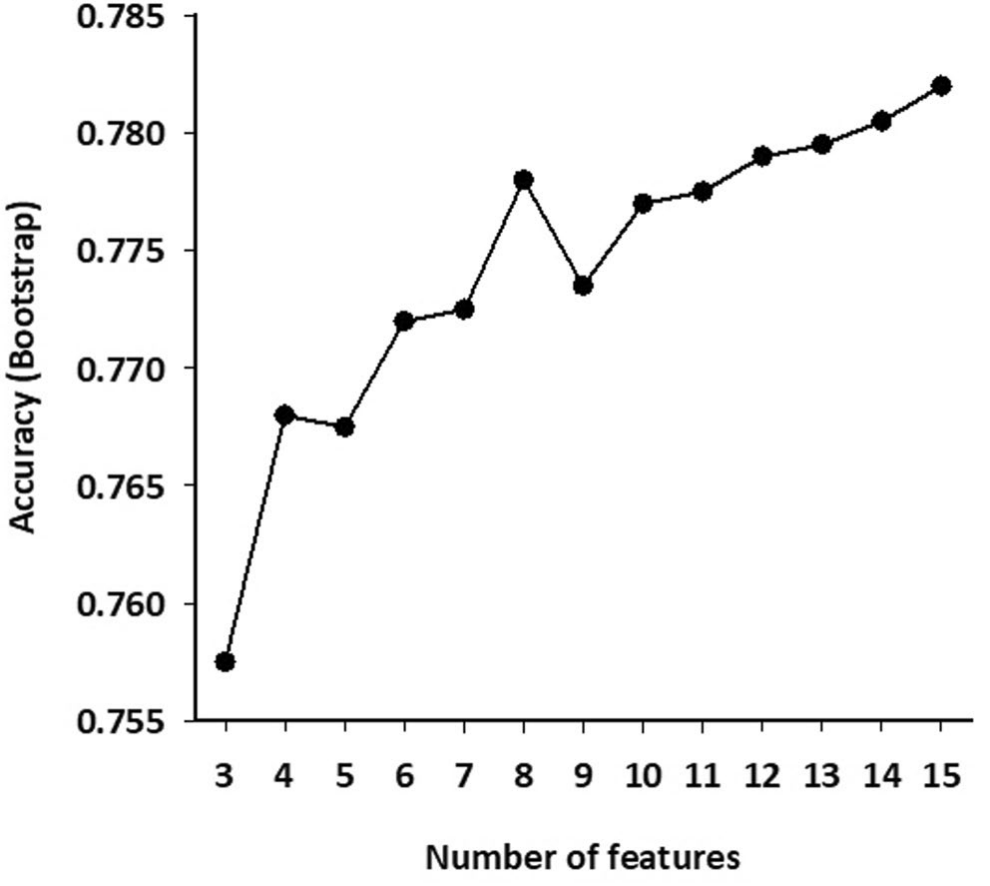
Results of the recursive feature elimination (RFE) variable selection procedure for the training dataset

To eliminate ineffective features and reduce redundancy, we performed variable selection before training the classification models. This process employed the Recursive Feature Elimination (RFE) method using the *caret* package [21]. To ensure the model’s generalizability, we utilized a naïve Bayes classifier alongside bootstrap resampling with 25 repetitions. Figure 7 illustrates the accuracy achieved based on the number and combination of features. The results indicate that using all 15 features yields the highest explained variance, justifying their inclusion in the final model.

### Estimation of feature importance in classification models

**Fig. 8:**
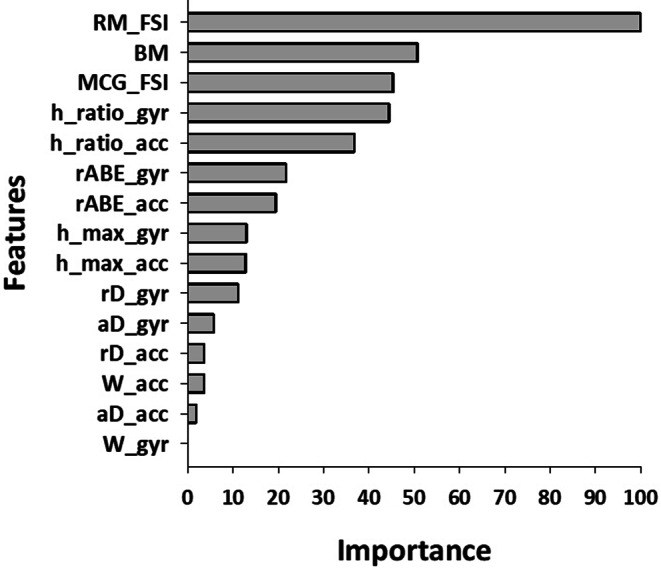
Feature importance in the selected final model

Feature importance in the final model was assessed using the *varImp* function from the *caret* package. This method evaluates the significance of each feature based on its contribution to the AUC metric. The importance scores range from 0 to 100, with 100 representing the feature that has the most substantial impact on the model’s predictions. Figure 8 displays the feature importance of the final Random Forest model used in this study.

**Table 4:** Characteristics of each subject and estimated REIs by 5 classification models

Subject	Age, yr	Sex	BMI, Kg/m^2^	AHI	ODI, 3%	Estimated REI				
						LogR	RF	GBM	KNN	MLP
Train_01	51	M	24.9	23.5	18.6	40.6	25.8	30.0	32.9	27.4
Train_02	55	M	24.7	3.1	2.2	9.9	4.1	16.1	9.4	10.3
Train_03	51	M	36.6	19.5	17.4	27.0	18.1	21.0	18.0	17.2
Train_04	37	M	22.2	7.7	6.3	22.4	7.4	0.0	0.0	0.0
Train_05	50	M	25.1	6.2	5.4	6.8	7.3	10.0	6.7	7.7
Train_06	57	M	26.0	11.8	10.1	15.8	13.1	16.8	15.7	19.0
Train_07	57	M	28.4	37.0	31.1	19.1	37.2	28.1	25.7	19.7
Train_08	39	M	27.9	62.6	55.6	68.4	62.0	64.5	65.5	64.3
Train_09	47	M	24.5	46.6	40.9	68.3	59.5	60.2	59.6	62.5
Train_10	21	F	26.8	10.3	9.1	12.7	11.2	2.6	16.4	11.0
Train_11	53	M	25.9	52.6	46.1	47.2	57.1	58.6	56.4	52.0
Train_12	45	M	27.1	2.7	2.6	17.0	2.6	5.3	6.3	3.6
Train_13	55	M	26.2	9.0	7.2	12.4	8.6	14.6	5.8	3.0
Train_14	48	M	32.1	44.8	38.8	52.1	39.1	34.4	43.2	36.1
Train_15	51	M	23.3	37.7	34.1	24.9	43.1	46.7	39.8	42.1
Train_16	46	M	30.3	33.8	30.8	54.6	42.3	55.6	52.0	58.5
Train_17	51	M	26.9	17.9	14.7	24.3	22.8	26.5	26.0	23.4
Train_18	73	M	24.9	22.7	17.7	19.5	14.3	14.7	14.0	11.6
Train_19	58	F	26.3	4.0	3.3	36.4	7.2	33.2	32.8	33.3
Train_20	53	M	25.3	21.1	17.9	18.6	24.0	29.9	25.1	27.3
Train_21	45	M	26.6	10.2	8.7	23.0	13.7	27.2	19.9	18.5
Train_22	67	F	24.9	9.6	7.6	13.1	7.3	16.1	8.7	13.2
Train_23	38	M	24.2	27.3	23.7	32.9	22.2	35.5	34.8	36.4
Train_24	51	M	29.2	14.7	12.3	1.1	17.8	14.8	11.9	5.7
Train_25	31	M	20.5	19.2	15.6	16.2	22.9	18.7	14.0	14.2
Train_26	46	M	24.3	7.6	6.0	3.2	7.2	10.0	5.7	5.8
Train_27	61	M	23.0	21.9	18.8	52.4	23.9	40.6	43.3	37.7
Train_28	58	M	32.8	14.1	12.2	32.6	10.4	0.5	3.0	17.9
Train_29	62	M	22.3	31.7	26.5	17.4	22.2	20.7	19.8	17.8
Train_30	23	F	19.6	0.9	0.1	6.0	1.6	5.9	3.1	4.1
Train_31	38	F	22.5	2.2	1.3	6.6	3.6	8.5	3.0	3.5
Train_32	59	F	19.8	4.7	2.7	17.2	4.5	10.8	9.0	3.9
Train_33	38	M	21.7	10.6	9.0	1.3	14.3	14.4	7.8	6.1
Train_34	50	M	22.6	6.1	4.6	30.1	7.6	27.5	23.2	27.2
Train_35	26	M	18.6	8.5	5.9	8.6	11.2	9.0	6.3	5.8
Train_36	63	M	27.0	74.0	69.1	93.4	72.7	84.4	76.7	85.6
Train_37	52	M	25.4	0.9	0.6	25.5	1.8	9.7	9.7	5.6
Train_38	48	M	25.3	17.0	14.8	42.1	20.4	24.8	27.5	31.1
Train_39	49	M	25.2	4.7	3.8	9.6	5.8	3.2	3.9	0.0
Train_40	43	M	30.7	28.6	24.6	30.1	26.9	26.4	25.9	22.3
Train_41	54	M	25.2	9.6	7.5	6.9	9.9	14.3	12.0	15.4
Test_01	47	M	34.3	19.6	19.2	44.7	18.3	24.1	31.3	21.7
Test_02	45	M	25.8	5.9	4.7	3.7	10.4	6.7	4.2	5.9
Test_03	58	M	26.2	43.2	37.1	23.2	37.5	34.1	36.7	32.1
Test_04	38	M	28.9	1.8	2.1	0.0	0.0	0.0	0.0	0.0
Test_05	29	M	41.6	106.1	99.3	112.8	92.6	94.4	96.8	96.8
Test_06	74	M	29.1	2.9	2.5	8.6	27.5	29.6	40.6	23.8
Test_07	67	M	24.9	72.8	65.6	85.2	97.4	93.0	96.6	93.7
Test_08	39	M	24.2	10.2	8.0	21.6	0.6	0.0	9.6	0.0
Test_09	76	M	30.1	15.7	14.0	30.9	32.5	34.6	32.2	25.7
Test_10	55	M	27.2	31.2	26.2	58.3	38.4	51.6	48.0	45.1
Test_11	58	M	25.2	24.7	19.7	18.6	21.3	19.2	15.9	15.9
Test_12	46	M	24.9	10.5	8.6	39.1	8.3	5.4	10.9	13.2
Test_13	48	M	30.8	9.4	8.8	0.0	7.6	0.0	0.0	0.0
Test_14	41	M	24.3	8.1	7.1	29.1	14.3	12.2	14.8	19.9
Test_15	58	F	26.0	12.7	10.6	35.9	12.9	8.1	22.8	13.9
Test_16	49	M	21.9	4.4	3.1	34.4	5.1	0.0	10.0	13.8
Test_17	47	M	24.1	48.9	41.5	50.7	50.6	54.2	54.7	48.0
Test_18	53	M	23.3	34.4	29.5	21.7	34.7	33.3	29.8	29.5
Test_19	55	M	21.1	6.2	4.2	18.9	13.0	10.8	12.8	10.7
Test_20	40	M	28.3	20.5	17.1	41.1	17.5	19.9	27.3	27.1

BMI, body mass index; AHI, apnea-hypopnea index; ODI 3%, 3% oxygen desaturation index; REI, respiratory event index; LogR, logistic regression; RF, Random Forest; GBM, Gradient Boosting Machine; KNN, k-Nearest Neighbor; MLP, Multilayer Perceptron

## Data Availability

The datasets generated during and/or analyzed during the current study are available from the corresponding author on reasonable request and with permission of Takaoka Clinic.

## Abbreviations

AASM: American Association of Sleep Medicine
AHI: Apnea-hypopnea index
AI: Apnea index
AUC: Area under the curve
BMI: Body mass index
CAI: Central apnea index
CI: Confidence interval
CPAP: Continuous positive airway pressure
BM: Body movement
FSI: Frequency stability index
GCG: Gyrocardiogram
HI: Hypopnea index
HSAT: Home sleep apnea testing
IMU: Inertial measurement unit
IQR: Inter quartile rage
MAI: Mixed apnea index
MCG: Mechanocardiogram
NPA: Negative predictive accuracy
OAI: Obstructive apnea index
PPA: Positive predictive accuracy
PPG: Photo-plethysmograms
PSG: Polysomnography
RE: Respiratory event
REI: Respiratory event index
RFE: Recursive feature elimination
RM: Respiratory wrist motion
RMS: Scalar time series of respiratory wrist motion; RM
SCG: Seismocardiogram
ROC: Receiver operating characteristic

## References

[1] Gharibeh T, Mehra R (2010) Obstructive sleep apnea syndrome: natural history, diagnosis, and emerging treatment options. Nat Sci Sleep 2:233–255. 10.2147/nss.S6844 23616712 10.2147/NSS.S6844 PMC3630950

[2] Morsy NE, Farrag NS, Zaki NFW, Badawy AY, Abdelhafez SA, El-Gilany AH, El Shafey MM, Pandi-Perumal SR, Spence DW, BaHammam AS (2019) Obstructive sleep apnea: personal, societal, public health, and legal implications. Rev Environ Health 34(2):153–169. 10.1515/reveh-2018-0068 31085749 10.1515/reveh-2018-0068

[3] Peppard PE, Young T, Barnet JH, Palta M, Hagen EW, Hla KM (2013) Increased prevalence of sleep-disordered breathing in adults. Am J Epidemiol 177(9):1006–1014. 10.1093/aje/kws342 23589584 10.1093/aje/kws342 PMC3639722

[4] Benjafield AV, Ayas NT, Eastwood PR, Heinzer R, Ip MSM, Morrell MJ, Nunez CM, Patel SR, Penzel T, Pépin JL, Peppard PE, Sinha S, Tufik S, Valentine K, Malhotra A (2019) Estimation of the global prevalence and burden of obstructive sleep apnoea: a literature-based analysis. Lancet Respir Med 7(8):687–698. 10.1016/s2213-2600(19)30198-5 31300334 10.1016/S2213-2600(19)30198-5 PMC7007763

[5] Faria A, Allen AH, Fox N, Ayas N, Laher I (2021) The public health burden of obstructive sleep apnea. Sleep Sci 14(3):257–265. 10.5935/1984-0063.20200111 35186204 10.5935/1984-0063.20200111 PMC8848533

[6] Punjabi NM (2008) The epidemiology of adult obstructive sleep apnea. Proc Am Thorac Soc 5(2):136–143. 10.1513/pats.200709-155MG 18250205 10.1513/pats.200709-155MG PMC2645248

[7] Pires GN, Arnardóttir ES, Islind AS, Leppänen T, McNicholas WT (2023) Consumer sleep technology for the screening of obstructive sleep apnea and snoring: current status and a protocol for a systematic review and meta-analysis of diagnostic test accuracy. J Sleep Res 32(4):e13819. 10.1111/jsr.13819 36807680 10.1111/jsr.13819

[8] Chen Y, Wang W, Guo Y, Zhang H, Chen Y, Xie L (2021) A single-center validation of the Accuracy of a photoplethysmography-based Smartwatch for Screening Obstructive Sleep Apnea. Nat Sci Sleep 13:1533–1544. 10.2147/nss.S323286 34557047 10.2147/NSS.S323286 PMC8453177

[9] Kim MW, Park SH, Choi MS (2022) Diagnostic performance of Photoplethysmography-based Smartwatch for Obstructive Sleep Apnea. J Rhinol 29(3):155–162. 10.18787/jr.2022.00424 39664308 10.18787/jr.2022.00424 PMC11524370

[10] Zhou G, Zhou W, Zhang Y, Zeng Z, Zhao W (2023) Automatic monitoring of obstructive sleep apnea based on multi-modal signals by phone and smartwatch. Annu Int Conf IEEE Eng Med Biol Soc 2023:1–4. 10.1109/embc40787.2023.10340237 38083356 10.1109/EMBC40787.2023.10340237

[11] Zhou G, Zhao W, Zhang Y, Zhou W, Yan H, Wei Y, Tang Y, Zeng Z, Cheng H (2024) Comparison of OPPO Watch Sleep Analyzer and Polysomnography for Obstructive Sleep Apnea Screening. Nat Sci Sleep 16:125–141. 10.2147/nss.S438065 38348055 10.2147/NSS.S438065 PMC10860396

[12] Cinar Bilge P, Keskintıg Fatma E, Cansu S, Haydar S, Deniz K, Alisher K, Sibel C, Ulufer C, Zuhal A, Ibrahim O (2024) Scanning of obstructive sleep apnea syndrome using smartwatch: a comparison of smartwatch and polysomnography. J Clin Neurosci 119:212–219. 10.1016/j.jocn.2023.12.009 38141437 10.1016/j.jocn.2023.12.009

[13] Borsky M, Serwatko M, Arnardottir ES, Mallett J (2022) Toward Sleep Study automation: detection evaluation of respiratory-related events. IEEE J Biomed Health Inf 26(7):3418–3426. 10.1109/jbhi.2022.3159727 10.1109/JBHI.2022.3159727 35294367

[14] Venek V, Kranzinger S, Schwameder H, Stöggl T (2022) Human Movement Quality Assessment Using Sensor Technologies in recreational and Professional sports: a scoping review. Sens (Basel) 22(13). 10.3390/s22134786 10.3390/s22134786 PMC9269395 35808282

[15] Liang W, Wang F, Fan A, Zhao W, Yao W, Yang P (2023) Extended application of Inertial Measurement Units in Biomechanics: from activity recognition to Force Estimation. Sens (Basel) 23(9):4229. 10.3390/s23094229 10.3390/s23094229 PMC10180901 37177436

[16] Bhongade A, Gupta R, Gandhi TK, Ap P (2023) A portable low-cost respiration rate Measurement System for Sleep Apnea Detection. Annu Int Conf IEEE Eng Med Biol Soc 2023:1–5. 10.1109/embc40787.2023.10340446 38083784 10.1109/EMBC40787.2023.10340446

[17] Hayano J, Adachi M, Sasaki F, Yuda E (2024) Quantitative detection of sleep apnea in adults using inertial measurement unit embedded in wristwatch wearable devices. Sci Rep 14(1):4050. 10.1038/s41598-024-54817-z 38374225 10.1038/s41598-024-54817-z PMC10876631

[18] Berry RB, Albertario CL, Harding SM, Uoyd RM, Plante DT, Quan SF, Troester MM, Vaughn BV (2018) The AASM Manual for the Scoring of Sleep and Association events: rules, terminology and technical specifications, Version 2.5. American Academy of Sleep Medicine, Darien, IL

[19] Santucci F, Lo Presti D, Massaroni C, Schena E, Setola R (2022) Precordial vibrations: a review of Wearable Systems, Signal Processing Techniques, and Main Applications. Sens (Basel) 22(15). 10.3390/s22155805 10.3390/s22155805 PMC9370957 35957358

[20] Ode KL, Shi S, Katori M, Mitsui K, Takanashi S, Oguchi R, Aoki D, Ueda HR (2022) A jerk-based algorithm ACCEL for the accurate classification of sleep-wake states from arm acceleration. iScience 25(2):103727. 10.1016/j.isci.2021.103727 35106471 10.1016/j.isci.2021.103727 PMC8784328

[21] Kuhn M (2008) Building Predictive models in R using the Caret Package. J Stat Softw 28(5):1–26. 10.18637/jss.v028.i0527774042

[22] Campbell AJ, Neill AM (2011) Home set-up polysomnography in the assessment of suspected obstructive sleep apnea. J Sleep Res 20(1 Pt 2):207–213. 10.1111/j.1365-2869.2010.00854.x 20561173 10.1111/j.1365-2869.2010.00854.x

[23] Dingli K, Coleman EL, Vennelle M, Finch SP, Wraith PK, Mackay TW, Douglas NJ (2003) Evaluation of a portable device for diagnosing the sleep apnoea/hypopnoea syndrome. Eur Respir J 21(2):253–259. 10.1183/09031936.03.00298103 12608438 10.1183/09031936.03.00298103

[24] Massie F, Van Pee B, Bergmann J (2022) Correlations between home sleep apnea tests and polysomnography outcomes do not fully reflect the diagnostic accuracy of these tests. J Clin Sleep Med 18(3):871–876. 10.5664/jcsm.9744 34710039 10.5664/jcsm.9744 PMC8883090

[25] Jagielski JT, Bibi N, Gay PC, Junna MR, Carvalho DZ, Williams JA, Morgenthaler TI (2023) Evaluating an under-mattress sleep monitor compared to a peripheral arterial tonometry home sleep apnea test device in the diagnosis of obstructive sleep apnea. Sleep Breath 27(4):1433–1441. 10.1007/s11325-022-02751-7 36441446 10.1007/s11325-022-02751-7

[26] Heneghan C, Chua CP, Garvey JF, de Chazal P, Shouldice R, Boyle P, McNicholas WT (2008) A portable automated assessment tool for sleep apnea using a combined Holter-Oximeter. Sleep 31(10):1432–1439 18853941 PMC2572749

[27] Hayano J, Watanabe E, Saito Y, Sasaki F, Fujimoto K, Nomiyama T, Kawai K, Kodama I, Sakakibara H (2011) Screening for obstructive sleep apnea by cyclic variation of heart rate. Circ Arrhythm Electrophysiol 4(1):64–72. 10.1161/CIRCEP.110.958009 21075771 10.1161/CIRCEP.110.958009

[28] Westenberg JN, Petrof BJ, Noel F, Zielinski D, Constantin E, Oskoui M, Kaminska M (2021) Validation of home portable monitoring for the diagnosis of sleep-disordered breathing in adolescents and adults with neuromuscular disorders. J Clin Sleep Med 17(8):1579–1590. 10.5664/jcsm.9254 33739260 10.5664/jcsm.9254 PMC8656910

[29] Agatsuma T, Fujimoto K, Komatsu Y, Urushihata K, Honda T, Tsukahara T, Nomiyama T (2009) A novel device (SD-101) with high accuracy for screening sleep apnoea-hypopnoea syndrome. Respirology 14(8):1143–1150. 10.1111/j.1440-1843.2009.01627.x 19818056 10.1111/j.1440-1843.2009.01627.x

[30] Sadek I, Heng TTS, Seet E, Abdulrazak B (2020) A New Approach for detecting Sleep Apnea using a contactless Bed Sensor: comparison study. J Med Internet Res 22(9):e18297. 10.2196/18297 32945773 10.2196/18297 PMC7532465

[31] Hayano J, Yamamoto H, Tanaka H, Yuda E (2024) Piezoelectric rubber sheet sensor: a promising tool for home sleep apnea testing. Sleep Breath 28(3):1273–1283. 10.1007/s11325-024-02991-9 38358413 10.1007/s11325-024-02991-9 PMC11196299

[32] Hayano J, Yamamoto H, Nonaka I, Komazawa M, Itao K, Ueda N, Tanaka H, Yuda E (2020) Quantitative detection of sleep apnea with wearable watch device. PLoS ONE 15(11):e0237279. 10.1371/journal.pone.0237279 33166293 10.1371/journal.pone.0237279 PMC7652322

[33] Kapur VK, Auckley DH, Chowdhuri S, Kuhlmann DC, Mehra R, Ramar K, Harrod CG (2017) Clinical practice Guideline for Diagnostic Testing for Adult Obstructive Sleep Apnea: an American Academy of Sleep Medicine Clinical Practice Guideline. J Clin Sleep Med 13(3):479–504. 10.5664/jcsm.6506 28162150 10.5664/jcsm.6506 PMC5337595

[34] Léger D, Stepnowsky C (2020) The economic and societal burden of excessive daytime sleepiness in patients with obstructive sleep apnea. Sleep Med Rev 51:101275. 10.1016/j.smrv.2020.101275 32169792 10.1016/j.smrv.2020.101275

[35] Hayano J, Yuda E (2021) Night-to-night variability of sleep apnea detected by cyclic variation of heart rate during long-term continuous ECG monitoring. Ann Noninvasive Electrocardiol 27(2):e12901. 10.1111/anec.12901 34661952 10.1111/anec.12901 PMC8916582

